# *Lactobacillus curvatus* UFV-NPAC1 and other lactic acid bacteria isolated from *calabresa*, a fermented meat product, present high bacteriocinogenic activity against *Listeria monocytogenes*

**DOI:** 10.1186/s12866-019-1436-4

**Published:** 2019-03-20

**Authors:** Nathália Parma Augusto Castilho, Monique Colombo, Leandro Licursi de Oliveira, Svetoslav Dimitrov Todorov, Luís Augusto Nero

**Affiliations:** 1 Departamento de Veterinária, Universidade Federal de Viçosa, Campus UFV, Viçosa, MG 36570-900 Brazil; 2 Departamento de Biologia Geral, Universidade Federal de Viçosa, Campus UFV, Viçosa, MG 36570-900 Brazil; 3 Faculdade de Ciências Farmacêuticas, Departamento de Alimentos e Nutrição Experimental, Universidade de São Paulo, Av. Prof. Lineu Prestes, 580, Bloco 1, Cidade Universitária, São Paulo, SP 05508-000 Brazil

**Keywords:** *Calabresa*, Bacteriocin, Lactic acid bacteria, *Listeria monocytogenes*, *Lactobacillus curvatus*

## Abstract

**Background:**

Bacteriocins produced by lactic acid bacteria (LAB) can be considered as viable alternatives for food safety and quality, once these peptides present antimicrobial activity against foodborne pathogens and spoilage bacteria. Fermented foods, such as artisanal sausages and cured meats, are relevant sources of LAB strains capable of producing novel bacteriocins, with particular interest by the food industry.

**Results:**

Three LAB strains (firstly named as *Lactobacillus curvatus* 12, *L. curvatus* 36 and *Weissella viridescens* 23) were obtained from *calabresa* by presenting promising bacteriocinogenic activity, distinct genetic profiles (rep-PCR, RAPD, bacteriocin-related genes) and wide inhibitory spectrum. Among these strains, *L. curvatus* 12 presented higher bacteriocin production, reaching 25,000 AU/mL after incubation at 25, 30 and 37 °C and 6, 9 and 12 h. Partially purified bacteriocins from *L. curvatus* 12 kept their inhibitory activity after elution with isopropanol at 60% (*v*/v). Bacteriocins produced by this strain were purified by HPLC and sequenced, resulting in four peptides with 3102.79, 2631.40, 1967.06 and 2588.31 Da, without homology to known bacteriocins.

**Conclusions:**

LAB isolates obtained from *calabresa* presented high inhibitory activity. Among these isolates, bacteriocins produced by *L. curvatus* 12, now named as *L. curvatus* UFV-NPAC1, presented the highest inhibitory performance and the purification procedures revealed four peptides with sequences not described for bacteriocins to date.

**Electronic supplementary material:**

The online version of this article (10.1186/s12866-019-1436-4) contains supplementary material, which is available to authorized users.

## Background

Lactic acid bacteria (LAB) can be considered as a major component of the microbiota of fermented meats, playing an important role to develop specific flavors and textures [1]. Despite these technological features, LAB has a biopreservative role in fermented meats, due to the production of antimicrobial compounds, such as organic acids and bacteriocins [1, 2]. Bacteriocins are protein compounds that present a variable spectrum of antimicrobial activity, usually against closely related species to the producer strains [3]. Numerous bacteriocins produced by LAB species have been already described and they are well known by their activity against spoilage bacteria and foodborne pathogens [3].

Nowadays, several studies have been reporting on isolation of bacteriocinogenic LAB from different sources, in order to investigate their potential as natural biopreservatives in different food products. Thus, the present study aimed to isolate bacteriocinogenic LAB strains from artisanal meats produced in Minas Gerais state, Brazil, and to characterize their produced bacteriocins towards their antimicrobial features and structure.

## Results

### Screening of bacteriocinogenic LAB

LAB counts in the examined samples were 5.3 × 10^6^ colony forming units per gram (CFU/g) for *choriço*, 6.4 × 10^6^ CFU/g for bacon and *calabresa* and 5.6 × 10^7^ CFU/g for *lombo defumado*. A total of 94 LAB isolates was obtained from the tested samples and selected due to the presence of inhibition halo against *Listeria monocytogenes* 72. After the confirmatory assays, 17 of them presented bacteriocinogenic activity. These isolates were obtained from *calabresa*, being confirmed as LAB (Gram positive, catalase negative).

Repetitive element palindromic-Polymerase Chain Reaction (rep-PCR) and Random Amplified Polymorphic DNA (RAPD) allowed the determination of five genetic profiles, leading to the selection of five representative LAB strains. Based on Basic Local Alignment Search Tool (BLAST) analysis, the sequencing of 16S rRNA allowed the identification of *Lactococcus garvieae* (one strain, firstly named as *L. garvieae* 32, with 100% of identity with other *L. garvieae* strains in GenBank), *Lactobacillus curvatus* (two strains, firstly named as *L. curvatus* 12 and *L. curvatus* 36, with 99 and 75%, respectively, of identity with other *L. curvatus* strains in GenBank) and *Weissella viridescens* (two strains, firstly named as *W. viridescens* 23 and *W. viridescens* 31, both with 99% of identity with other *W. viridescens* strains in GenBank).

### Characterization of the bacteriocinogenic potential

Results for amplification of bacteriocin related genes in the tested LAB strains are presented in Table 1*. L. curvatus* strains presented similar genetic profiles regarding bacteriocin related genes: both presented amplified products for *sakTA* and *sakTB*, and *L. curvatus* 36 also presented positive results for *plaW* and *plaS*. *W. viridescens* strains also presented similar genetic profiles, with positive results for *ped*, *plaW*, and *sakTA* and *sakTB*; *plaS* was also recorded in *W. viridescens*31. *L. garvieae* 32 presented positive results only for *entP* and *sakTA*.

**Table 1:** Results for bacteriocin related genes in five bacteriocinogenic strains of lactic acid bacteria isolated from *calabresa*, obtained by PCR (positive: +; negative: -)

Target bacteriocin	Bacteriocinogenic strain				
	*L. garvieae* 32	*L. curvatus* 12	*L. curvatus* 36	*W. viridescens* 23	*W. viridescens* 31
*entA*	–	–	–	–	–
*entP*	+	–	–	+	–
*entB*	–	–	–	–	–
*entL50B*	–	–	–	–	–
*ped*pro	–	–	–	+	+
*nis*	–	–	–	–	–
*plaW*	–	–	+	+	+
*plaNC8*	–	–	–	–	–
*plaS*	–	–	+	–	+
*sakGA1*	–	–	–	–	–
*sakGA2*	–	–	–	–	–
*sakX*	–	–	–	–	–
*sakA*	–	–	–	–	–
*sakQ*	–	–	–	–	–
*sakP*	–	–	–	–	–
*sakTA*	–	+	+	+	+
*sakTB*	+	+	+	+	+

Obs.: primers and PCR conditions are detailed in Additional file 1: Table S1

The inhibitory spectrum of the selected bacteriocinogenic LAB is presented in Table 2. Three of the tested strains (*L. curvatus* 12, *L. curvatus* 36 and *W. viridescens* 23) presented a high potential in inhibiting the Gram positive targets usually associated with human infections and usual focus of bacteriocin studies, like *L. monocytogenes* (from different serotypes), *Enterococcus* spp. and *Staphylococcus*; low frequencies of inhibition were observed for LAB (except *Enterococcus* spp.), and no inhibitory activity was recorded against the tested Gram negative bacteria. *L. garvieae* 32 and *W. viridescens* 31 presented inhibitory activity against only one *L. monocytogenes* strain (Table 2). Considering the results observed until this step, *L. curvatus* 12, *L. curvatus* 36 and *W. viridescens* 23 were selected for further analyses regarding their bacteriocinogenic activity and *L. monocytogenes* 72 was selected as the target organism.

**Table 2:** Frequencies of inhibitory activity of the cell free supernatant of bacteriocinogenic lactic acid bacteria isolated from *calabresa* against different targets, assessed by the spot-on-the-lawn methodology

Genus	Species or Serotype	n	Producer strain				
			*L. garvieae* 32	*L. curvatus* 12	*L. curvatus* 36	*W. viridescens* 23	*W. viridescens* 31
*Listeria*	*L. monocytogenes* 1/2a	1	0	1	1	1	0
	*L. monocytogenes* 1/2b	1	0	1	1	1	0
	*L. monocytogenes* 1/2c	12	0	11	11	11	0
	*L. monocytogenes* 4b	3	1	3	3	3	1
	*L. monocytogenes* (not serotyped)	8	0	6	6	6	0
	*L. innocua*	1	0	1	1	1	0
	*L. welshimeri*	1	0	1	1	1	0
*Enterococcus*	*E. faecium*	3	0	3	3	3	0
	*E. faecalis*	4	0	3	3	3	0
	*E. durans*	1	0	1	1	1	0
	*E. hirae*	1	0	1	1	1	0
*Staphylococcus*	*S. aureus*	2	0	1	1	1	0
*Lactococcus*	*L. lactis* subsp. *cremoris*	1	0	1	1	1	0
*Lactobacillus*	*L. sakei*	1	0	1	1	1	0
	*L. casei*	4	0	1	1	0	0
	*L. acidophilus*	1	0	0	0	0	0
	*L. nagelli*	1	0	0	0	0	0
	*L. harbinensis*	2	0	0	0	0	0
	*L. fermentum*	1	0	0	0	0	0
	*L. plantarum*	3	0	1	1	0	0
*Pediococcus*	*P. pentosaceus*	1	0	0	0	0	0
	*P. acidilactici*	1	0	0	0	0	0
*Weissella*	*W. paramesenteroides*	2	0	0	0	0	0
*Corynebacterium*	*C. vitaeruminis*	1	0	1	1	0	0
	*P. aeruginosa*	1	0	0	0	0	0
	*P. fluorescens*	1	0	0	0	0	0
*Escherichia*	*E. coli*	2	0	0	0	0	0
*Salmonella*	*S.* Thyphimurium	2	0	0	0	0	0
	*S.* Typhi	1	0	0	0	0	0

Treatment of the cell free supernatant (CFS) with different enzymes resulted in complete inactivation of the inhibitory activity (Table 3). None of the tested CFS lost their antimicrobial activity after treatment with α-amylase, lipase or catalase, confirming that the studied antimicrobial peptides have not carbohydrate or lipid moiety in their structures, neither antimicrobial activity is result of production of hydrogen peroxide (H_2_O_2_) (Table 3). pH, temperature and chemicals promoted different patterns of interference on the inhibitory activity of the CFS from the tested LAB strains (Table 3). Temperature did not affect substantially the inhibitory activity of the CFS of the studied strains: only for *L. curvatus* 36 was observed a decrease on the inhibitory activity after treatments at 40, 60 and 80 °C (Table 3). Regarding tested chemicals, CFS of *L. curvatus* 36 lost its inhibitory activity after treatment with sodium chloride (NaCl) and Tween 80, while the CFS of the other strains kept their inhibitory activity at different levels (Table 3).

**Table 3:** Effects of different substances and incubation conditions on the inhibitory activity of the cell free supernatant of bacteriocinogenic lactic acid bacteria isolated from *calabresa* against *L. monocytogenes* 72

effect	substance/condition	concentration	producer strain		
			*L. curvatus* 12	*L. curvatus* 36	*W. viridescens* 23
Enzymes^a^	trypsin	0.1 mg/mL	AI	AI	AI
	proteinase K	0.1 mg/mL	AI	AI	AI
	papain	0.1 mg/mL	AI	AI	AI
	pepsin	0.1 mg/mL	AI	AI	AI
	protease	1 mg/mL	AI	AI	AI
	α-amilase	1 mg/mL	I	I	I
	lipase	1 mg/mL	I	I	I
	catalase	1 mg/mL	I	I	I
pH^a^	3		AI	AI	I
	5		AI	AI	I
	7		I	I	I
	8		I	AI	I
	10		I	AI	I
Temperature^b^	7 °C for 30 min		3200	12,800	25,600
	25 °C for 30 min		3200	25,600	25,600
	37 °C for 30 min		25,600	25,600	25,600
	40 °C for 30 min		25,600	12,800	25,600
	60 °C for 30 min		25,600	12,800	25,600
	80 °C for 30 min		25,600	6400	25,600
Chemicals^b^	NaCl	10 mg/mL	3200	0	200
	EDTA	10 mg/mL	200	800	800
	Tween 80	10 mg/mL	200	0	400

^a^effect of enzymes was assessed by the spot-on-the-lawn method. “AI” indicates absence of inhibition, “I” indicates inhibition; ^b^ effects of pH, temperature and chemicals were assessed by a quantitative assay, and results expressed as arbitrary units per mL

### Optimization of bacteriocin production

**Fig. 1:**
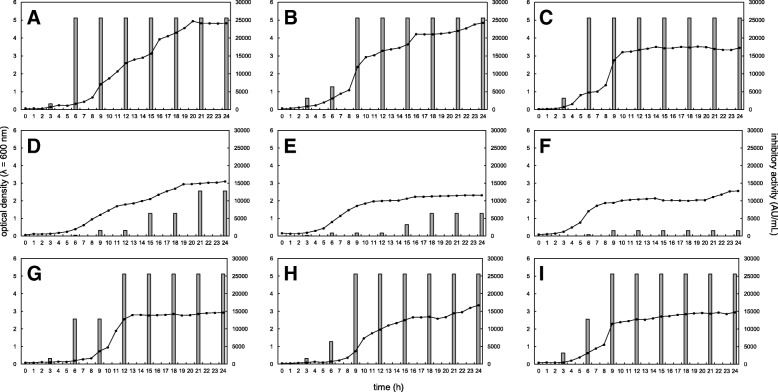
Growth (optical density at λ = 600 nm) and inhibitory activity (arbitrary units per mL) of *Lactobacillus curvatus* 12 (**a**, **b**, **c**), *L. curvatus* 36 (**d**, **e**, **f**) and *Weissella viridescens* 23 (**g**, **h**, **i**) cultivated in MRS broth and incubated at 25 (**a**, **d**, **g**), 30 (B, E, H) and 37 °C (**c**, **f**, **i**)

Growth and bacteriocin production by the bacteriocinogenic strains cultured in de Man, Rogosa and Sharpe (MRS) broth at 25, 30 and 37 °C are presented in Fig. 1. All strains presented similar growth pattern, independently of the incubation temperature. Also, all strains presented a similar pattern of acidification, monitored by changes of pH in the tested temperatures, ranging from 6.0 to 4.0 along the incubation (data not shown). Independently of the level of bacteriocin production, inhibitory activity was stable in all tested conditions and no decrease of it was recorded in the end of stationary phase (Fig. 1).

Table 4 shows the interference of variations on MRS composition on the inhibitory activity of the tested bacteriocinogenic strains. Based on the observed data, *L. curvatus* 12 was the strains that presented less influence of the tested variation on its inhibitory activity, being decreased only at some pH variations (2, 8 and 10), some carbohydrates sources (lactose, sacarose, mannitol and raffinose) and absence of manganese sulphate (MnSO_4_) and Tween 80. The inhibitory activity of *W. viridescens* 23 was totally inhibited when the strain was cultured at pH 10 and 12, and in absence of MnSO_4_, and *L. curvatus* 36 was highly affected by the MRS composition, once only the variations on MgSO4 did not result in a loss or decrease of its inhibitory activity.

**Table 4:** Effects of variations on MRS broth on inhibitory activity of bacteriocinogenic lactic acid bacteria isolated from *calabresa* against *L. monocytogenes* 72, assessed by a quantitative assay

Effect	MRS variant	Concentration (mg/mL)	Bacteriocinogenic strain		
			*L. curvatus* 12	*L. curvatus* 36	*W. viridescens* 23
Control	MRS	–	25,600	25,600	25,600
pH	2	–	200	0	1600
	4	–	25,600	200	1600
	6	–	25,600	25,600	25,600
	8	–	1600	0	1600
	10	–	12,800	0	0
	12	–	25,600	0	0
Carbo-hydrate	lactose	20	1600	0	25,600
	sacarose	20	800	0	25,600
	mannitol	20	3200	0	800
	fructose	20	25,600	0	25,600
	dextrose	20	25,600	25,600	25,600
	maltose	20	25,600	0	25,600
	raffinose	20	12,800	100	25,600
Organic nitrogen	peptone	25	25,600	0	25,600
	meat extract	25	25,600	0	25,600
	yeast extract	25	25,600	100	25,600
	peptone + meat extract	12.5 + 12.5	25,600	100	12,800
	peptone + yeast extract	15 + 7.5	25,600	200	25,600
	meat extract + yeast extract	15 + 7.5	25,600	100	25,600
	peptone + meat extract + yeast extract	10 + 10 + 5	25,600	25,600	25,600
Chemicals	KH_2_PO_4_	0	25,600	100	12,800
		2	25,600	25,600	25,600
		5	25,600	100	25,600
		10	25,600	0	25,600
	MgSO_4_	0	25,600	25,600	100
		0.1	25,600	25,600	25,600
		0.5	25,600	25,600	100
	MnSO_4_	0	1600	25,600	0
		0.05	25,600	25,600	25,600
		0.2	25,600	0	100
	Sodium acetate	0	25,600	0	25,600
		5	25,600	25,600	25,600
		10	25,600	100	25,600
	Tri-ammonium citrate	0	25,600	0	25,600
		2	25,600	25,600	25,600
		5	25,600	100	25,600
	Tween 80	0	12,800	0	12,800
		1	25,600	25,600	25,600
		2	25,600	100	25,600
		5	25,600	200	25,600

These obtained data (Fig. 1, Table 4) highlighted the better inhibitory performance of *L. curvatus* 12 when compared to the other tested LAB strains, *L. curvatus* 36 and *W. viridescens* 23.

### Inhibition of *L. monocytogenes* 72 growth by LAB CFS

**Fig. 2:**
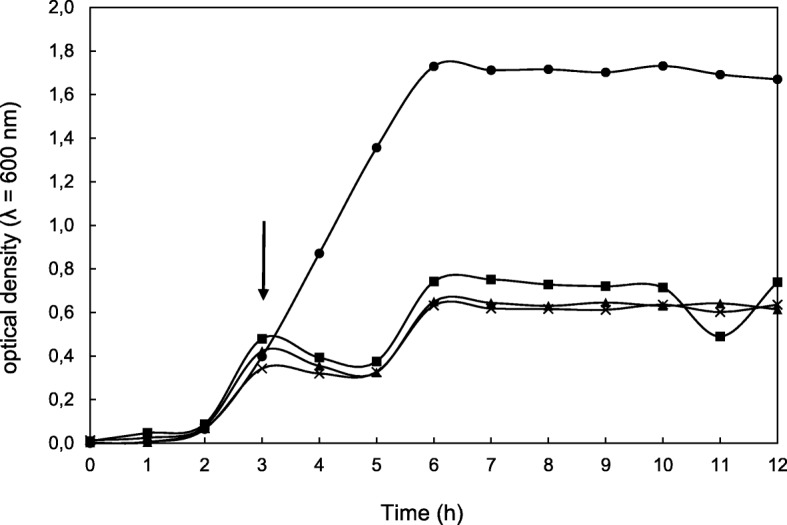
Growth curves of *Listeria monocytogenes* 72 inoculated in brain heart infusion and incubated at 37 °C for 12 h, and added with cell free supernatant (CFS) of bacteriocinogenic lactic acid bacteria strains at 3 h of incubation (arrow). Control (●), adding of *Lactobacillus curvatus* 12 CFS (▲), *L. curvatus* 36 CFS (×) and *Weissella viridescens* 23 CFS (■)

The inhibitory effects of the produced bacteriocins on *L. monocytogenes* 72 were assessed in two steps: first, in target cells at stationary phases, and second, in the beginning of their log growth phase (after 3 h). Considering the first approach, CFS of *L. curvatus* 12 determined complete inhibition of *L. monocytogenes* 72. CFS from *L. curvatus* 36 and *W. viridescens* 23 were able to reduce *L. monocytogenes* 72 populations to approximately 10^2^ CFU/mL. Based on the second approach, Fig. 2 shows the effects of the CFS when added to the target culture after 3 h of incubation: CFS produced by the bacteriocinogenic strains were able to reduce substantially the growth of *L. monocytogenes* 72.

**Fig. 3:**
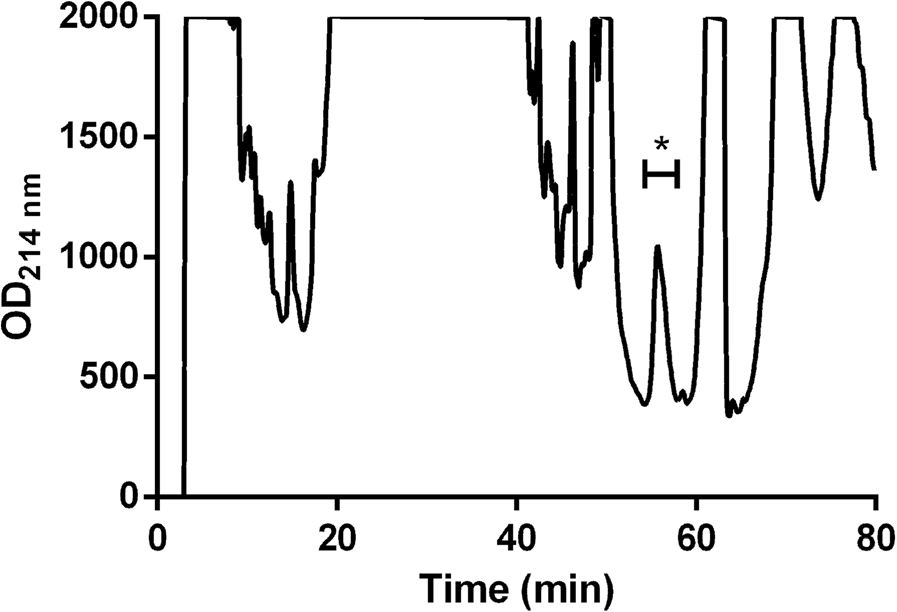
Chromatogram of the purified bacteriocins produced by *Lactobacillus curvatus* 12 (C4 reversed-phase HPLC). Asterisk indicates the peak with inhibitory activity against *L. monocytogenes* 72, with retention time of 56.27 min

Despite presenting similar inhibitory activity in the beginning of growth phase of *L. monocytogenes* 72 (Fig. 3), the obtained data in the assay that evaluated the inhibitory activity of the CFS in the stationary phase of the target indicated the better performance of *L. curvatus* 12 when compared to *L. curvatus* 36 and *W. viridescens* 23.

### Partial purification and purification of bacteriocins

After the partial purification procedures from the CFS produced by the bacteriocinogenics strains, inhibitory activity was observed after elution with 60% isopropanol. However, inhibitory activity was also observed with 40 and 80% isopropanol, at lower levels when compared to 60% isopropanol (data not shown).

**Fig. 4:**
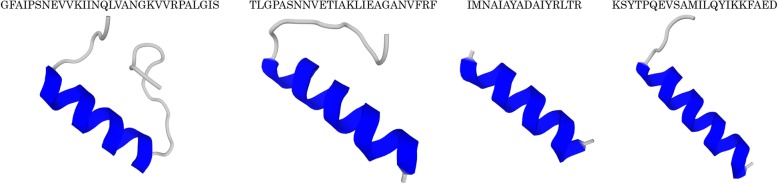
Predicted structures of four peptides produced by *Lactobacillus curvatus* 12 (C4 reversed-phase HPLC) designed using Pepfold3 (http://bioserv.rpbs.univ-paris-diderot.fr)

After precipitation with ammonium sulphate, the purified proteins produced by *L. curvatus* 12 presented inhibitory activity against *L. monocytogenes* 72. The extract submitted to a high-performance liquid chromatography (HPLC) using a C4 column also presented inhibitory activity against *L. monocytogenes* 72 and resulted in one partially isolated peak (Fig. 3). The sequencing results indicated four different peptides: GFAIPSNEVVKIINQLVANGKVVRPALGIS (3102.79 Da), TLGPASNNVETIAKLIEAGANVFRF (2631.40 Da), IMNAIAYADAIYRLTR (1967.06 Da) and KSYTPQEVSAMILQYIKKFAED (2588.31 Da). The predicted third structures of the obtained peptide sequences are presented in Fig. 4.

## Discussion

Despite the current demand for artisanal products from animal origin by consumers, they can offer some microbiological hazards due to natural presence of pathogens, especially from raw meats, demanding good sanitary practices during production, curing and storage. *Calabresa* and *chouriço* are produced with raw meats (bovine and swine) and seasoning, stuffed in intestinal casings and subjected to smoking and curing; *lombo defumado* is usually produced with the pork tenderloin, added with cure salts, and subjected to smoking and curing; and *bacon* is usually produced with pork belly, added with cure salts and subjected to smoking and mild curing [1]. Then, the production of these meat products requires a rigorous hygienic control to avoid bacterial contamination and growth of undesirable microorganisms [1]. However, these meat products also contains a rich autochthonous microbiota composed mainly by LAB, and many of them are capable of producing inhibitory substances against different microorganisms, mainly *L. monocytogenes* and other foodborne pathogens and spoilage bacteria [4]. LAB constitute a relevant part of the initial microbiota in meats and their populations easily grow during cold storage, even when these products are packed under modified atmosphere or vacuum, and after processing of fermented products [1]. *Lactobacillus* and *Lactococcus* species are among the main members of the meat lactic microbiota [1], as well as *Weissella* species [5], in accordance with the results obtained in the present study with fermented meats. Isolates belonging to these genera were already isolated from fermented meats and described as bacteriocinogenic [6–8], demonstrating the potential of these foods as sources of novel LAB strains with biopreservative potential.

The variable pattern of results for bacteriocin related genes was already expected, once they can be easily transferred among different LAB in a same food matrix with a complex microbiota (Table 1). In the present study we identified that *L. curvatus* 36, *W. viridescens* 23, and *W. viridescens* 31 carry the *plaW* gene suggesting that these isolates may produce a similar antimicrobial peptide. This gene was already described in a *L. plantarum* strain [9], and plantaricins W and S, both produced by *Lactobacillus*, were described as bacteriocins composed by two peptides [10, 11]. Also, *L. curvatus* 36 and *W. viridescens* 31 presented *plaS* (Table 1), indicating their potential in producing the two-antimicrobial peptides, and *sakTA* and *sakTB* were detected in almost all isolates (Table 1). The presence of bacteriocin-related genes does not necessarily indicate the expression of these peptides, specially enterocin-related genes [12]: horizontal gene transfer mechanisms can explain the variety of genes and the production of multiple bacteriocins by the same culture [13]. *Enterococcus* strains have mechanisms of genetic exchange explaining the variable presence of enterocins related genes in wild strains [13]. Expression of pediocin PA-1 is associated to the presence of *pedA*, *pedB*, *pedC* and *pedD* in the bacteriocinogenic strain, most probably located in a plasmid [14]. The obtained results for *ped* operon related genes indicate the presence of the full machinery for pediocin PA-1 production in the *Weissella* strains (Table 1), suggesting their ability to produce this bacteriocin.

The tested LAB isolates presented a wide inhibitory spectrum (Table 2). Assessing the inhibitory spectrum of bacteriocinogenic strains is required to evaluate the potential application of the producer strains as biopreservatives and probiotics, due to the natural biodiversity of target strains in food and gastrointestinal tract [3]. The low frequencies of LAB inhibition (Table 2) demonstrate the potential usage of the bacteriocinogenic strains as biopreservatives in fermented foods.

Confirmation of proteinaceous nature of bacteriocins is essential step in characterization of new antimicrobial peptides. CFS of selected LAB isolates added to enzymes resulted in no inhibition, confirming their proteinaceous natures (Table 3). α-amylase, lipase or catalase did not influence the inhibitory activity of LAB isolates (Table 3); testing such substances are important to identify possible components of bacteriocins structure, as well as to identify inhibitory activity due to the production of other antimicrobial substances produced by the producer strains. After treatment with α-amylase, bacteriocin ST63BZ lost its inhibitory activity [15], as observed for leuconocin S [16], indicating that their activity was associated with glycosylation of the active peptide.

Inhibitory activity of LAB isolates was variable after pH, temperature and chemicals treatment (Table 3). pH played an important role on the inhibitory activity of the studied bacteriocins: *L. curvatus* 12 CFS kept its antimicrobial activity at pH values higher than 7.0, *L. curvatus* 36 CFS presented inhibitory activity only after pH 7.0, and *W. viridescens* 23 CFS kept its inhibitory activity after all tested pH values. Some studies reported a higher inhibitory activity of bacteriocins when subjected to acids when compared to alkaline: nisin is known by its sensitivity to alkaline pH values [17] and leucocin F10 kept its inhibitory activity after acid treatments, but it was sensitive to pH 7.0 and 9.0 [18]. Regarding temperature, once bacteriocins are small peptides, they are usually thermostable, as previously described [3] and observed in the present study (Table 3). However, lactocin NK24 lost around 90% of its inhibitory activity after treatment at 121 °C for 15 min [19].

Resistance to different chemicals is important to lead the technological application of bacteriocins, as well as to guide the adoption of different laboratorial procedures for further characterization of them. The chemical urea, Tween 20, Tween 80 and ethylene-diamine-tetra-acetic acid (EDTA), when added at different concentrations, determined different impacts on the inhibitory activity of four bacteriocins produced by LAB isolated from *boza*, pediocin ST18 and enterocin EJ97 [20–22].

When compared to other tested LAB isolates, *L. curvatus* 12 reached higher levels of growth (Fig. 1). Also, this isolate and *W. viridescens* 23 presented high levels of production of bacteriocin after 6 or 9 h of incubation, independently of the temperature; *L. curvatus* 36 presented higher production of bacteriocin when cultured at 25 °C, compared to other incubation temperatures, and only after 21 h of incubation (Fig. 1). Similar profile of bacteriocin production was already reported for several antimicrobial peptides expressed by different strains of *Lactobacillus* spp. [6, 9].

Based on variations on MRS composition, tested LAB isolates presented a variable pattern of inhibitory activity (Table 4). pH plays an important role in bacteriocin stability (Table 3) and production (Table 4) and many studies describe the relevance of its initial value in the culture media considered for bacteriocin production by bacteriocinogenic strains [4, 23]. *L. curvatus* 12 was able to produce bacteriocins at low pH (Table 4), despite the produced substances being susceptible to these conditions (Table 3); *L. curvatus* 36 produced bacteriocins at high levels only in neutral pH (Table 4), which corresponds to pH for its bacteriocins stability (Table 3); despite *W. viridescens* 23 CFS were stable at all tested pH (Table 3), the production of bacteriocins occurred only and acid and neutral pH values (Table 4). Production of bacteriocins can be related to the metabolism of growth medium, and it is not necessarily related to microbial growth. Different patterns of production of some bacteriocins can be associated to variations of different carbohydrate and organic nitrogen sources [4, 23], as peptone can improve the production of plantaricin 423 [24] and meat extract for pediocin PA-1/AcH [25]. Little is known about the influence of potassium ions on the bacteriocins production: high concentrations of K_2_HPO_4_ decreased the production of some bacteriocins [4], while plantaricin UG1 production was enhanced by it [26]. Tri-ammonium citrate was described as enhancer of bacteriocin ST8KF production [27], while the absence of magnesium sulphate (MgSO_4_) and MnSO_4_ determined a decrease on bacteriocin ST8KF production [27]. Tween 80 was also described as responsible to enhance the bacteriocin production at specific concentrations [4].

*L. monocytogenes* 72 was totally inhibited by the CFS from *L. curvatus* 12, while CFS from *L. curvatus* 36 and *W. viridescens* 23 reduced its populations at 10^2^ CFU/mL (data not shown). Considering a similar approach, bacteriocin HA-6111-2 produced by *Pediococcus acidilactici* was able to determine a complete inhibition of *E. faecium* HKLHS populations [28]. However, when the CFS from *L. curvatus* 12 was added to the log growth of *L. monocytogenes* 72, the recorded inhibition was similar when compared to the effects of the CFS from the other tested LAB isolates (Fig. 2). Adding the bacteriocin DF04Mi at 3200 arbitrary units per mL (AU/mL) to a 3 h-old culture of *L. monocytogenes* resulted in growth inhibition for at least 12 h [29]. Other bacteriocins, such as the ones produced by *E. faecium* ST5Ha and *P. acidilactici* HA-6111-2, presented a similar behavior [15, 28].

After partial purification of bacteriocins from CFS of the tested LAB isolates, the fraction of 60% isopropanol presented higher inhibitory activity when compared to other fractions. Based on a similar approach, bacteriocin ST44AM also presented inhibitory activity after partial purification on SePakC18 at 60% isopropanol [14], while plantaricin ST31 presented this activity at 40% isopropanol [30]. Considering the obtained results, the bacteriocins produced by *L. curvatus* 12 were partially purified in a single chromatographic step and the peak with inhibitory activity revealed four structurally similar peptides (Figs. 3 and 4). X Zhu, Y Zhao, Y Sun and Q Gu [31] used a four-step purification method, including XAD-2, cation-exchange chromatography, gel chromatography and HPLC and successfully obtained a pure peptide; these authors observed that the purified bacteriocin was resistant to N-terminal sequencing and the sequencing showed no homology with other known bacteriocins. MS Barbosa, SD Todorov, I Ivanova, JM Chobert, T Haertlé and BDGM Franco [6] purified two bacteriocins achieve by the three-step procedure, such as cation-exchange followed by sequential hydrophobic-interaction and reversed-phase chromatography, and they observed two peaks in the final chromatogram of each bacteriocin tested; these procedures resulted in successful purification of both bacteriocins. The obtained sequences were subjected to a further analysis on Bactibase (http://bactibase.hammamilab.org/) and the identified peptides showed no similarity with any family of bacteriocins.

*L. curvatus* was already characterized as usual member of meat microbiota [1, 32] and often added in fermented meats as a starter culture responsible for acidification and inhibition of undesirable bacteria [33]. Despite these known beneficial features for meat fermented products, the obtained data demonstrated the bacteriocinogenic potential of *L. curvatus* 12, now named as *L. curvatus* UFV-NPAC1, guiding further studies to demonstrate its application to improve the quality and safety of meat products. Also, the partially-purified or purified bacteriocins can be potentially employed as biopreservatives in fermented foods that the technological properties of *L. curvatus* UFV-NPAC1 are not required.

## Conclusions

LAB isolates obtained from *calabresa* presented high inhibitory potential, and some strains were able to produce bacteriocins with potential application in the food industry as biopreservatives, specially towards *L. monocytogenes*. Among these isolates, bacteriocins produced by *L. curvatus* 12, now named as *L. curvatus* UFV-NPAC1, presented the highest inhibitory performance and the purification procedures revealed four peptides with sequences not described for bacteriocins to date.

## Methods

### Samples and bacteriocinogenic LAB screening

Artisanal meat products (*lombo defumado*, *calabresa*, bacon and *chouriço*) were purchased at the Central Market of Belo Horizonte (Minas Gerais, Brazil) and portions of 25 g were aseptically transferred to 225 mL of sterile peptone water 0.1% (*w*/*v*) (SPW, Oxoid Ltd., Basingstoke, England), homogenized and ten-fold diluted with SPW. Aliquots of 100 μL from selected dilutions were surface plated in duplicates on MRS agar plates (Becton, Dickinson and Company - BD, Franklin Lakes, NJ, USA), overlaid with agar-agar 1% (w/v) (BD), and incubated at 37 °C for 48 h. After incubation, colonies were enumerated and results were expressed as CFU/g.

Plates containing individual colonies were selected and overlaid with 5 mL of brain heart infusion (BHI, BD) supplemented with 0.75% (w/v) agar (BD) inoculated with *L. monocytogenes* 72, serotype 4b, previously isolated from beef [34], at approximately 10^6^ CFU/mL. Plates were incubated at 37 °C for 24 h and colonies with clear inhibition zones were transferred to MRS broth (BD) and incubated at 37 °C for 24 h [35]. The obtained isolates were subjected to Gram staining and tested for catalase production using hydrogen peroxide at 3% (*v*/v). Gram positive and catalase negative isolates were transferred to MRS broth (BD), incubated at 37 °C overnight, and the obtained cultures were stored at − 20 °C with glycerol at 20% (v/v).

Aliquots of the stock cultures (*n* = 94) were transferred to MRS broth (BD) and incubated at 37 °C for 24 h. The obtained cultures were centrifuged (10,000×*g*, 4 °C, 15 min) and the CFS were adjusted to pH 6.0 with sodium hydroxide (NaOH) 1 M and heated at 80 °C for 10 min. The treated CFS were subjected to the spot-on-the lawn assay to identify the inhibitory activity of strains: aliquots of 10 μL of CFS were spotted on the surface of plates containing BHI agar (BD) or MRS agar (BD) previously inoculated with *L. monocytogenes* 72 (10^6^ CFU/mL). Plates were incubated at 37 °C for 24–48 h, and inhibition halos larger than 2 mm were indicative of potential bacteriocinogenic activity of the CFS producer isolate.

The isolates that presented potential bacteriocinogenic activity were selected (*n* = 17) and subjected to DNA extraction by using ZR Fungal/Bacterial DNA Kit (Zymo Research, Irvine, CA, USA). DNA concentrations were determined by using NanoDrop (Thermo Fisher Scientific, Waltham, MA, USA) and used for fingerprinting by rep-PCR using primer GTG_5_ [36], and RAPD-PCR using primers OPL01, OPL02, OPL04, OPL05, OPL14 and OPL20 [4].

Based on rep-PCR and RAPD profiles, five strains were selected and their DNA were subjected to PCR to amplify a region of 16S rRNA [37] and sequencing (Center for Human Genome Studies, Institute of Biomedical Sciences, University of São Paulo, São Paulo, SP, Brazil). The obtained sequences were subjected to analysis by using the BLAST (GenBank, National Center for Biotechnology Information, Bethesda, MD, USA) for identification.

### Characterization of the bacteriocinogenic potential

The selected five strains (firstly named as *L. garvieae* 32, *L. curvatus* 12, *L. curvatus* 36, *W. viridescens* 23 and *W. viridescens* 31) were subjected to DNA extraction as described above and to PCR reactions to detect genes related to the production of the following bacteriocins: enterocins A, P, B and L50B, pediocin PA-1, nisin, plantaricins W, NC8 and S, and sakacins GA-1, GA-2, X, A, Q, P, Tα and Tβ [8, 10, 11, 38–43]. Primer sequences and PCR conditions are described in the Additional file 1: Table S1.

The CFS of the selected strains was tested for antimicrobial activity against a panel of 64 strains, composed by Gram positive (*Listeria* spp. = 27, *Enterococcus* spp. = 9, *Staphylococcus aureus* = 2, *Lactococcus lactis*: 1, *Lactobacillus* spp. = 13, *Pediococcus* spp. = 2, *Weissella paramesenteroides* = 2, *Corynebacterium vitaeruminis* = 1) and Gram negative (*Pseudomonas* spp. = 2, *Escherichia coli* = 2, *Salmonella* spp. = 3). All target strains are property of the Universidade Federal de Viçosa (UFV, Viçosa, MG, Brazil) or American Type Culture Collection (ATCC, Manassas, VA, USA). The spot-on-the-lawn method, as described above, was used for this characterization.

Based on the obtained results, three LAB strains were selected (*L. curvatus* 12, *L. curvatus* 36 and *W. viridescens* 23) for further inhibitory assays. The stability of the treated CFS from these selected strains was assessed after treatment with enzymes [22]: aliquots of 1 mL of the CFS were added with trypsin (0.1 mg/mL), proteinase K (0.1 mg/mL), papain (0.1 mg/mL), pepsin (0.1 mg/mL), protease (1 mg/mL), α-amylase (1 mg/mL), lipase (1 mg/mL) and catalase (1 mg/mL) (previously diluted with their adequate buffers, and all from Sigma-Aldrich, St. Louis, MI, USA), incubated at 30 °C for 30 min, heated at 90 °C for 5 min, and cooled at 25 °C. pH was assessed by adding hydrochloric acid (HCl) 1 M or NaOH 1 M to CFS in order to reach pH values of 3.0, 5.0, 7.0, 8.0 and 10.0, and incubated at 30 °C for 1 h. Temperature was assessed by incubating CFS aliquots at 7, 25, 37, 40, 60 and 80 °C for 30 min. Also, CFS were supplemented with NaCl, EDTA and Tween 80 (all at end concentrations of 10 mg/mL, Sigma-Aldrich) and incubated at 30 °C for 30 min. CFS subjected to treatments with enzymes and pH were tested for inhibitory activity against *L. monocytogenes* 72 by the spot-on-the lawn assay, as described above. CFS subjected to treatments of temperature and chemicals were subjected to a quantitative assay to verify the inhibitory activity of the produced bacteriocins [22]: samples were subjected to two-fold serial dilution with phosphate buffer (100 mM, pH 6.5), spotted (10 μL) on the surface of plates containing BHI agar (BD) inoculated with *L. monocytogenes* 72, and incubated at 37 °C for 24 h; Bacteriocin activity was expressed as AU/mL, corresponding to the reciprocal of the highest dilution having a detectable halo of inhibition (higher than 2 mm). As control, the inhibitory activity of the untreated CFS was assessed using the same protocols.

### Optimization of bacteriocin production

Studied strains were inoculated in MRS broth (BD), and incubated at 25, 30 and 37 °C for 24 h. In each 1 h, aliquots of cultures were obtained and subjected to spectrophotometry at 600 nm (UV-M51, Bell Photonics do Brasil, São Paulo, SP, Brazil) and pH measuring (W3B, Bell). In each 3 h, CFS of the cultures were obtained and treated (as described above), and subjected to a quantitative assay to verify the inhibitory activity against *L. monocytogenes* 72 of the produced bacteriocins (as described above).

Also, the selected strains were transferred to 10 mL of MRS broth (BD), incubated at 37 °C for 24 h, and their cells were obtained by centrifugation (10,000×*g* for 5 min) and washed two times with sterile 0.85% NaCl (*w*/*v*). Then, cells were suspended with 10 mL of 0.85% NaCl (w/v), and aliquots de 100 μL were used for inoculation in MRS broth with modified characteristics [29]: pH (MRS broth [BD] adjusted to 2.0, 4.0, 6.0, 8.0, 10.0 and 12.0, by using NaOH 1 M or HCl 1 M), carbohydrate source (lactose, sacarose, dextrose, D-mannitol, fructose, maltose or raffinose, all at 20.0 g/L [*w*/*v*] and from Sigma-Aldrich, instead of glucose), organic nitrogen source (tryptone at 20 mg/mL, meat extract at 20 mg/mL, yeast extract at 20 mg/mL, tryptone at 12.5 mg/mL and meat extract at 7.5 mg/mL, tryptone at 12.5 mg/mL and yeast extract at 7.5 mg/mL, meat extract at 10 mg/mL and yeast extract at 10 mg/mL, and tryptone at 10 mg/mL and meat extract at 5 mg/mL and yeast extract at 5 mg/mL, all from Sigma-Aldrich), and other chemicals (dipotassium phosphate, K_2_HPO_4_, at 0, 5.0 and 10.0 mg/mL; MgSO_4_ at 0, 0.1 and 0.5 mg/mL; MnSO_4_ at 0, 0.05 and 0.2 mg/mL; sodium acetate at 0, 5.0 and 10.0 mg/mL; tri-ammonium citrate at 0, 2.0 and 5.0 mg/mL; Tween 80 at 0, 1.0, 2.0 and 5.0 mg/mL; glycerol at 0, 0.5, 1.0, 2.0, 5.0 and 10.0 mg/mL). The MRS variations were assessed individually. Cultures were incubated at 37 °C for 24 h, when the bacteriocin activity was determined against *L. monocytogenes* 72 by a quantitative assay (as described above).

### Inhibition of *L. monocytogenes* 72 growth by LAB CFS

*L. monocytogenes* 72 was selected as target strain to evaluate the inhibitory effect of the CFS produced by the selected LAB strains. *L. monocytogenes* 72 was cultured in 10 mL of BHI (BD) for 18 h at 37 °C, when the cells were centrifuged (10,000×*g* for 5 min), washed two times with sterile 0.85% NaCl (*w*/*v*) and resuspended in 10 mL of NaCl 0.85% (w/v). Equal volumes of *L. monocytogenes* 72 suspensions and CFS (obtained as described above) of the bacteriocinogenic strains were mixed and incubated at 37 °C for 1 h. Then, *L. monocytogenes* 72 was ten-fold diluted (NaCl 0.85%, w/v) and pour plated in BHI agar (BD), followed by incubation at 37 °C for 24, when colonies were enumerated and results were expressed as CFU/mL. Target cell suspensions mixed with NaCl 0.85% (w/v) were considered as controls.

Also, the target strain was inoculated in BHI (BD) at approximately 10^6^ CFU/mL. Then, aliquots of 100 mL of the inoculated BHI were distributed in four flasks and incubated at 37 °C for 12 h. After the initial 3 h of incubation, 20 mL of the CFS of the bacteriocinogenic strains were individually inoculated to the target cultures. A target culture without CFS adding was considered as growth control. In each hour, aliquots of the cultures were obtained and subjected to spectrophotometry at 600 nm (UV-M51, Bell) [44].

### Partial purification of bacteriocins

Bacteriocinogenic strains were cultured in 1000 mL of MRS (BD), at 37 °C for 24 h. CFS was obtained as described above. Proteins were precipitated by adding ammonium sulphate to 300 mL of the CFS, in order to obtain 40, 60 and 80% of saturation. The mixtures were stirred for 4 h in orbital shaker at 4 °C, centrifuged (20,000×*g*, 1 h, 4 °C), and the obtained pellet was re-suspended in 30 mL of phosphate buffer (PB, 100 mM, pH 6.5). Partial separation of proteins was performed by SepPakC_18_ hydrophobic column (Merck Millipore, Burlington, MA, USA), with a gradient of different concentrations of isopropanol (20, 40, 60, 80%) in PB (100 mM, pH 6.5). All obtained fractions were tested for bacteriocin activity by the spot-on-the-lawn method against *L. monocytogenes* 72, as described above. Fractions that presented bacteriocinogenic activity were selected and freeze dried. Before use, the dried material was resuspended in ultrapure water (MilliQ water, Merck Millipore).

### Bacteriocin purification

Based on previous results, *L. curvatus* 12 was selected for bacteriocin purification. *L. curvatus* 12 was inoculated in MRS broth (BD), incubated at 37 °C for 24 h and the CFS was obtained as described above. CFS was precipitated by adding ammonium sulphate 40%, incubated at 4 °C for 2 h, centrifuged (8000×g, 4 °C, 20 min) and the obtained pellet was re-suspended in MiliQ water (Merck Millipore). The obtained extract was tested for its inhibitory activity against *L. monocytogenes* 72 using the spot-on-the-lawn assay, as described above. The obtained extract was subjected to a HPLC using a C4 column (4.6 mm × 250 mm, Vidac) and mobile phase A (0.1% Formic acid in water) and B (0.1% Formic acid in acetonitrile). The system was equilibrated with 5% B and the absorbed substances were eluted with a linear gradient from 5 to 80% B. The fractions were collected and dried in SpeedVac (Thermo Fisher) for 1 h, and the pellet was re-suspended in MiliQ water (Merck Millipore) and tested for its inhibitory activity against *L. monocytogenes* 72 by the spot-on-the-lawn methodology, as described above.

HPLC fractions that presented inhibitory activity against *L. monocytogenes* 72 were dried. Then, the fractions were solubilized in 400 μL of 0.1% formic acid, and analyzed in a nanoscale liquid chromatography coupled to tandem mass spectrometry (nano LC-MS) using the nanoAcquity UPLC system (Waters, Milford, MA, USA). Briefly, aliquots of 1 μL of sample were injected and the gradient was linearly varied 2–30% B (*v*/v) in 59 min, 30–85% in 5 min at a flow rate of 0.3 μL/min in nanoAcquity UPLC BEH C18 column (1.7 μm, 130 Ǻ, 100 μm × 100 mm). The eluted peptides were automatically injected into a mass spectrometer model MAXIS 3G (Bruker Daltonics, Billerica, MA, USA), acting in online mode with a CaptiveSpray ionization source. Peptide analysis was performed using an appropriate method (IE_GCF_01-02-2017), with the drying gas flow rate of 3 L/min, temperature of ionization source of 150 °C and the transmission voltage of 2 kV.

The raw data were converted to a mass list and compared against the *Lactobacillus* protein bank, deposited in the Uniprot Consortium using the PEAKS application version 7.0 (Bioinformatics Solutions Inc., Waterloo, ON, Canada) [45]. The parameters used for the research were: the analyzed peptides not originating from enzymatic cleavage; error tolerance for the 20 ppm parental ion and for the 0.6 Da fragments; carbamidomethylation of cysteine as a fixed modification and oxidation of methionine as a variable modification; for the identification to be accepted, the result should contain at least one unique peptide and the false discovery rate (FDR) less than one. The obtained peptide sequences were subjected to a further analysis by using the software Pepfold3, in order to have their third structure predicted (http://bioserv.rpbs.univ-paris-diderot.fr).

## Additional file

Additional file 1: **Table S1.** PCR primers used for detection of bacteriocin-related genes in lactic acid bacteria isolated from *calabresa*, a fermented sausage. (DOCX 19 kb)

## Abbreviations

°C: Celsius degrees
Ǻ: Angstrom
ATCC: American Type Culture Collection
AU/mL: arbitrary units per mL
BLAST: Basic Local Alignment Search Tool
CFS: cell free supernatant
CFU/g or mL: colony forming units per gram or millilitre
Da: Dalton
DNA: deoxyribonucleic acid
EDTA: ethylene-diamine-tetra-acetic acid
FDR: false discovery rate
H2O2: hydrogen peroxide
HCl: hydrochloric acid
HPLC: High performance liquid chromatography
K2HPO4: dipotassium phosphate,
LAB: lactic acid bacteria
M: Molar
MgSO4: magnesium sulphate
mm: millimetre
MnSO4: manganese sulphate
MRS: de Man, Rogosa and Sharpe
NaCl: Sodium chloride
nano LC-MS: nanoscale liquid chromatography coupled to tandem mass spectrometry
NaOH: sodium hydroxide
PB: phosphate buffer
ppm: parts per million
RAPD: Random Amplified Polymorphic DNA
rep-PCR: repetitive element palindromic-Polymerase Chain Reaction
UFV: Universidade Federal de Viçosa
μL: microliter
μm: micrometre
