# Supplementary material for: Lactobacillus curvatus UFV-NPAC1 and other lactic acid bacteria isolated from calabresa, a fermented meat product, present high bacteriocinogenic activity against Listeria monocytogenes
Source: BMC Microbiol. 2019 Mar 20;19:63. doi: 10.1186/s12866-019-1436-4 (PMC6425648; doi:10.1186/s12866-019-1436-4)
Supplement: Supplementary file 1 — Table S1. PCR primers used for detection of bacteriocin-related genes in lactic acid bacteria isolated from calabresa, a fermented sausage. (DOCX 19 kb) [file 12866_2019_1436_MOESM1_ESM.docx]

Supplementary Table. Primers and PCR conditions for detection of bacteriocin related genes in five bacteriocinogenic lactic acid bacteria isolated from *calabresa*.

| Bacteriocin | Target gene | Primer | Sequence (5'-3') | Annealing (°C) | Size (bp) | Reference |
| --- | --- | --- | --- | --- | --- | --- |
| Enterocin A | *entA* | EntA - F | AAATATTATGGAAATGGAGTGTAT | 34 | 452 | du Toit et al., 2000 |
|  |  | EntA - R | GCACTTCCCTGGAATTGCTC |  |  |  |
| Enterocin P | *entP* | EntP- F | TATGGTAATGGTGTTTATTGTAAT | 41 | 216 | du Toit et al., 2000 |
|  |  | EntP- R | ATGTCCCATACCTGCCAAAC |  |  |  |
| Enterocin B | *entB* | EntB- F | GAAAATGATCACAGAATGCCTA | 41 | 159 | du Toit et al., 2000 |
|  |  | EntB- R | GTTGCATTTAGAGTATACATTTG |  |  |  |
| Enterocin L50B | *entL50* | EntL50B- F | STGGGAGCAATCGCAAAATTAG | 44 | 135 | du Toit et al., 2000 |
|  |  | EntL50B- R | ATTGCCCATCCTTCTCCAAT |  |  |  |
| Pediocin PA-1 | *ped*pro | PedA - F | CAAGATCGTTAACCAGTTT | 44 | 1238 | Todorov et al., 2016 |
|  |  | PedA- R | CCGTTGTTCCCATAGTCTAA |  |  |  |
| Nisin | *nis* | NisF | ATGAGTACAAAAGATTTCAACTT | 48 | 203 | Kruger et al., 2013 |
|  |  | NisR | TTATTTGCTTACGTGAACGC |  |  |  |
| Plantaricin W | *plaW* | PlanW- F | TCACACGAAATATTCCA | 41 | 165 | Holo et al., 2010 |
|  |  | PlanW- R | GGCAAGCGTAAGAAATAAATGAG |  |  |  |
| Plantaricin NC8 | *plaNC8* | PlanNC8- F | GGTCTGCGTATAAGCATCGC | 35 | 207 | Maldonado et al., 2003 |
|  |  | PlanNC8- R | AAATTGAACATATGGGTGCTTTAAATTCC |  |  |  |
| Plantaricin S | *plaS* | PlanS - F | GCCTTACCAGAGTAATGCCC | 45 | 450 | Stephens et al., 1998 |
|  |  | PlanS - R | CTGGTGATGCAATCGTTAGTTT |  |  |  |
| Sakacin GA-1 | *sakGA1* | SakGA1- F | TTAGAACTACACTGCTCGTG | 38 | 259 | Todorov et al., 2011 |
|  |  | SakGA1- R | TGGAAGAATGAGTACTTGTT |  |  |  |
| Sakacin GA-2 | *sakGA2* | SakGA2- F | CGTTACAACAGAACTTCAAG | 38 | 259 | Todorov et al., 2011 |
|  |  | SakGA2- R | TGGAAGAATGAGTACTTGTT |  |  |  |
| Sakacin X | *sakX* | SakX - F | AGCTATGAAAGGTATTGTCGGG | 62 | 156 | Macwana and Muriana, 2012 |
|  |  | SakX - R | TAAGATTTCCAGCCAGCAGC |  |  |  |
| Sakacin A | *sakA* | SakA- F | GAAWTRMMANCAATTAYMGGTGG | 55 | 150 | Dortu et al., 2008 |
|  |  | SakA- R | CAGCCGCTAATCATACCACC |  |  |  |
| Sakacin Q | *sakQ* | SakQ- F | GAARTWSYANCAATTADNGGTGG | 53 | 130 | Dortu et al., 2008 |
|  |  | SakQ- R | TACCACCAGCAGCCATTCCC |  |  |  |
| Sakacin P | *sakP* | SakP- F | ATGGAAAAGTTTATTGAATTA | 40 | 186 | Reminger et al., 1996 |
|  |  | SakP- R | TTATTTATTCCAGCCAGCGTT |  |  |  |
| Sakacin Tα | *sakTA* | SakT-α - F | TCGGTGGCTATACTGTCTAAACA | 58 | 160 | Macwana and Muriana, 2012 |
|  |  | SakT-α - R | TGTCCTAAAAATCCACCAATGC |  |  |  |
| Sakacin Tβ | *sakTB* | SakT-β - F | AAGAAATGATAGAAATTTTTGGAGG | 56 | 151 | Macwana and Muriana, 2012 |
|  |  | SakT-β - R | TGTGAAATCCAATCTTGTCCTG |  |  |  |
